# Acetaminophen administration in pediatric age: an observational prospective cross-sectional study

**DOI:** 10.1186/s13052-016-0219-x

**Published:** 2016-02-26

**Authors:** Riccardo Lubrano, Sara Paoli, Marco Bonci, Luigi Di Ruzza, Corrado Cecchetti, Raffaele Falsaperla, Piero Pavone, Nassim Matin, Giovanna Vitaliti, Isotta Gentile

**Affiliations:** Department of Pediatrics, La Sapienza University of Rome, Rome, Italy; Paediatrics Operative Unit, Grassi Hospital, Rome, Italy; Paediatrics Operative Unit, Policlinico Umberto I, Frosinone, Italy; Acute and Emergency Operative Unit, Bambino Gesù Paediatric Hospital, Rome, Italy; General Paediatrics and Acute and Emergency Paediatrics Operative Unit, Vittorio Emanuele University Hospital, Catania, Italy; University Medical Science of Teheran, University of Teheran, Teheran, Italy; AOU Policlinico-OVE, University of Catania, Via Plebiscito n. 628, 95100 Catania, Italy

**Keywords:** Children, Off label, Supratherapeutic dose, Acetaminophen, Prescription, Educational level

## Abstract

**Background:**

Parents often do not consider fever as an important physiological response and mechanism of defense against infections that leads to inappropriate use of antipyretics and potentially dangerous side effects. This study is designed to evaluate the appropriateness of antipyretics dosages generally administered to children with fever, and to identify factors that may influence dosage accuracy.

**Results:**

In this cross-sectional study we analyzed the clinical records of 1397 children aged >1 month and < 16 years, requiring a primary care (ambulatory) outpatient visit due to fever. We evaluated the number of children who had received >90 mg/kg/day of acetaminophen, the prescriber, the medication formula and the educational level of the caregiver who administered acetaminophen. Among those children included in our study, 74 % were administered acetaminophen for body temperature ≤ 38.4 °C. 24.12 % of children received >90 mg/kg/day of acetaminophen. Parents with university qualifications most commonly self-administered acetaminophen to their children, in a higher than standard dose. Self medication was also described in 60 % of children, whose acetaminophen was administered for temperatures < 38 °C. Acetaminophen over-dosage was also favored by the use of drug formulations as drops or syrup.

**Conclusions:**

Our study shows that preventive action should be taken regarding the use of acetaminophen as antipyretic drug in children in order to reduce the fever phobia and self-prescription, especially of caregivers with higher educational levels. It is also necessary to promote a more appropriate use of acetaminophen in those parents using drops or syrup formulations.

## Background

In recent years a fever phobia phenomenon [1] has been recognized among parents, who consider fever as a health danger instead of an important physiologic response and a mechanism of defense against infections [2, 3].

This phenomenon, associated with an increase in self-medication [4] has resulted in inappropriate use of antipyretics, amongst which the most commonly used is acetaminophen [5, 6].

The dosage of acetaminophen in children is assessed according to weight [7]; thus a wrong weight measurement or wrong perception of the child’s weight can lead to inappropriate medication dosing. Also, dosing based on age may cause errors in overweight or underweight children [8–10].

Actually, the appropriate dosage of acetaminophen in children is 10–15 mg/kg/dose administered every 4–6 h [11]. Higher doses of acetaminophen may be hepatotoxic, thus its administration should be performed carefully. Acetaminophen toxicity may be consequent to the administration of higher dosages both for over-dosages (in terms of quantity per dose) and more frequent standard doses (in terms of frequencies) of acetaminophen or excessive in numbers of administrations per day in 24 h, causing a daily overdose of > 90 mg/kg/day [11–17]. Other authors [14, 18] have described adverse effects even with doses of 75 mg/kg/day, however they were not able to clarify what conditions may be responsible for the onset of toxicity in some children, while the same doses have been shown not to be toxic for matched groups of patients. In regards, the Italian Society of Pediatrics suggest the daily maximal dose of acetaminophen of 80 mg/kg/day, defining toxic a dose of 150 mg/Kg [19].

The pediatric acute and emergency departments are privileged areas to observe how acetaminophen is used by caregivers in cases of fever. The study was performed on children admitted to the acute care and emergency departments of some Hospitals in the Lazio region. The objective of our study was to evaluate the appropriateness of the dosage of acetaminophen generally administered to children with fever, and the factors that may influence dosage accuracy.

## Methods

We performed an observational cross-sectional study in a period of six months, in accordance with STROBE guidelines [20] from November 2012 to April 2013.

The study setting is made of three health care services (General Paediatrics Operative Unit, Grassi Hospital of Rome, Policlinico Umberto I of Frosinone and General Paediatrics Operative Unit, Vittorio-Emanuele Hospital, Catania) affiliated to SIMEUP (Italian Society for Pediatric Emergency). Children aged between 1 month and 16 years, who required a primary care outpatient visit due to fever, to whom acetaminophen was administered by caregivers as antipyretic drug, from almost 24 h, were enrolled in this study.

In the pediatric emergency department, caregivers were asked to respond to a questionnaire inquiring on: the age and weight of the child, if acetaminophen was administered before entering the hospital, the dosage and frequency of administration, type of medication: liquid (syrup or drops) or solid (suppositories or tablets), the underlying body temperature that prompted the administration of medication, if other antipyretic drugs had been administered before, parent’s nationality, the parent giving the medication, level of education, and the name of the prescriber. At questionnaire administration, a healthcare physician explained how to fill the form to the parents.

Exclusion criteria were applied to the following categories:

- Children with fever to whom no antipyretic drug was administered
- Children with fever to whom acetaminophen was not administered as antipyretic
- Children taking antipyretics other than acetaminophen,
- Children with incomplete questionnaires,
- Children whose parents were of different nationalities (for example one Italian and one with a foreign nationality). These patients were excluded to reduce the potential for bias related to different cultural behaviors.

The primary outcome of the study was to evaluate the dosage of acetaminophen administered both as mg/kg/dose and as total daily quantity in mg/kg/day. Dosage of up to 15 mg/kg/dose (group A) was considered normal. Dosage of more than 15 mg/kg/dose (group B) was considered as supratherapeutic [11, 14]. Also the daily quantity (mg/kg/dose × n° of doses) was considered as not potentially dangerous if < 90 mg/kg/die (group C) or potentially dangerous if > 90 mg/kg/day (group D), [11].

Secondary outcomes were the temperature for which antipyretics were used and the factors that could influence medication’s dosages, such as:

- Educational level of the caregiver who administered acetaminophen to the child (elementary school, primary school, secondary school, university education)
- Type of prescription: medical or self-prescription
- Pharmaceutical formulation: liquid, drops or syrup, (DSy group) or solid, tablets or suppositories, (TSu group).
- Parents nationality, differentiating couples of parents as Italians (I) and Foreigners (F)

### Statistics

Data is analyzed with JMP 10 for mac.

Chi-square was used for differences between groups of categorical variables.

Wilcoxon test was used for analyzing the differences between different quantities of acetaminophen. Because estimating, initially, the approximation of population distribution to normal by Kolmogorov-Smirnov One-Sample Test and statistics for kurtosis and symmetry, it was asymmetrically distributed and non-parametric tests were used. Results for each studied group were expressed as median/3 rd quartile/1 st quartile

A *p* value of < 0.05 is considered as statistically significant.

Also, a contingency analysis was used to verify the distribution of patients with respect to the caregivers’ nationality and the daily dose of administered acetaminophen.

### Study approval

The protocol conforms to the ethical guidelines of the 1975 Declaration of Helsinki as revised in year 2000, and was approved by the institution’s ethical committee [21].

## Results

A total of 2108 children who were admitted to Acute and Emergency Departments because of fever were selected for this study. At baseline 203 children to whom no antipyretic drug was administered were excluded. Also 108 children, who used other antipyretic drugs and 95 children who assumed acetaminophen plus another different antipyretic drug were excluded. Parents of those children included in the study were asked to fill an evaluation questionnaire. After collecting questionnaires, 289 children were excluded as their parents did not fully fill the questionnaire. Sixteen other children were also excluded because their caregivers’ questionnaire showed that their parents were of different nationality. Thus, a total of 1397 questionnaires were analyzed. Median age of children was 72 months (with 3 rd quartile of 118 months and 1 st quartile of 48 months).

**Fig. 1:**
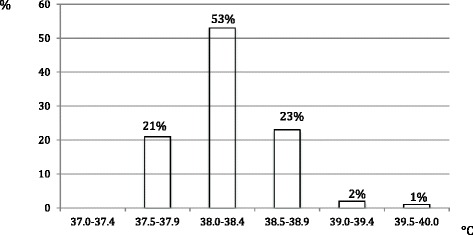
Body temperature to which the parents reported to have begun to treat the fever

Acetaminophen was administered in 74 % of cases to children with a body temperature below 38.4 °C (Fig. 1).

As shown in Table 1, the daily dose of acetaminophen was < 90 mg/kg/day (group C) in 1060 (75.88 %) cases and > 90 mg/kg/day (group D) in 337 (24.12 %) cases.

**Table 1:** Subgroups of the studied population accordingly to the total daily dose (group D > 90 mg/kg/day; Group C < 90 mg/kg/die) and the single daily dose (group A < 15 mg/kg/dose; group B > 15 mg/kg/dose;) of assumed acetaminophen

Group dose/day	N (%)	Medical prescription/self-prescription %/%	mg/kg/day	Sub-group dose	N (%)	mg/kg/dose	N. of doses	mg/kg/day
			Median			Median	Median	Median
			3 rd quartile			3 rd quartile	3 rd quartile	3 rd quartile
			1 st quartile			1 st quartile	1 st quartile	1 st quartile
Group D	337 (24.12)	9,82/14.3	107.14	<15 mg/kg/dose (Group A)	8 (2.37)	13.39	8.00	107.10
						14.76	8.00	118.10
			123.71			11.94	8.00	95.52
>90 mg/kg/day				>15 mg/kg/dose (Group B)	329 (97.63)	19.53	6.00	107.14
			97.61			22.73	6.00	123.97
						17.24	5.00	97.66
Group C	1060 (75.88)	75.88/0	58.20	<15 mg/kg/dose (Group A)	703 (66.32)	11.68	4.00	52.63
						13.44	6.00	68.18
			73.13			9.62	4.00	39.06
<90 mg/kg/day				>15 mg/kg/dose (Group B)	357 (33.68)	18.11	4.00	68.96
			44.78					
						20.00	4.00	76.92
						16.67	3.00	61.68

Group D vs C (mg/kg/day) *p* < 0.001

Group D: Sub-group A vs Sub-group

mg/kg/dose *p* < 0.0001; number of doses *p* < 0.0001; mg/kg/day p NS

Group C: Sub-group A vs Sub-group B

mg/kg/dose *p* < 0.0001; number of doses *p* < 0.0001; mg/kg/day *p* < 0.0001

mg/kg/dose for C vs D sub-groups

CA vs DB; DB vs CB; DA vs CB *p* < 0.0001 – DA vs CA p NS

Number of doses for C vs D sub-groups

CA vs DB; DB vs CB; DA vs CB; DA vs CA *p* < 0.0001

mg/kg/day for C vs D sub-groups

CA vs DB; DB vs CB; DA vs CB; DA vs CA *p* < 0.0001

Among those who assumed more than 90 mg/kg/day (group D), Acetaminophen was administered more than 15 mg/kg/dose in 97.63 %, while the remaining 2.37 % received less than 15 mg/kg/dose, with a higher frequency with respect to standard during the 24 h (Table 1).

Data on Table 2 shows that in group C (daily dose < 90 mg/kg/day), the educational level of the caregiver who administered acetaminophen to his/her child, was secondary education in 63 % cases, primary education in 33 % and an elementary education in 4 %, while in group D (daily dose > 90 mg/kg/day) was university degree in 71 % of cases and a secondary degree in 29 %. As a matter of fact Table 2 shows that the caregiver responsible for the administration of higher daily doses of acetaminophen is the one with a higher educational level (caregiver with a university degree: dose/day 121.73 ± 22.89 mg).

**Table 2:** Influence of the level of education on the total daily dose of administered acetaminophen

	Group C			Group D	
	<90 mg/kg/day			>90 mg/kg/day	
Level of education	Elementary school	Primary education	Secondary education	Secondary education	University education
Number	44	347	669	98	239
% in the group	4	33	63	29	71
% of the total population	3.15	24.84	47.88	7.01	17.11
Dose/day mg/kg/die	21.47/23.94/17.51	41.67/46.58/35.71	69.44/77.40/60.42	93.75/96.65/92.98	115.38/128.20/107.14

Dose/day = median/ 3 rd quartile/1 st quartile

*C* group C, *D* group D

*El* Elementary school, *P* primary education, *S* secondary education, *U* University education

El vs P; El vs S-C; El vs S-D; S-D vs U; P vs S-C; P vs S-D; P vs U; S-C vs S-D; S-D vs U all *p* < 0.0001

Acetaminophen was administered as tablets or suppositories (group TSu) in 87.97 % of children and drops or syrup (group DSy) in 12.03 %. The daily dose of acetaminophen (mg/kg/day) was significantly higher in those who assumed drops or syrup rather than those who assumed tablets or suppositories, both for children in group C (dose/day < 90 mg/kg/day) [the dose is expressed as median (3 rd quartile/1 sd quartile)], [DSy vs TSu: 69.81 (80.97/52.63) vs 57.69 (71.91/44.44) *p* < 0.003; DSy 5 %, TSu 95 %] and those in group D (dose/day > 90 mg/kg/die) [DSy vs TSu: 117.65 (133.33/100) vs 105.63 (116.28/96.38) *p* < 0.0001; DSy 35 %, TSu 65 %]. Moreover, odds ratio revealed a higher risk of acetaminophen overdose when drops and/or syrup formulations were used, *OR* = 0.089 (lower 95 % = 0.061 Upper 95 % =0.13)

Finally, acetaminophen was prescribed by a health professional for 85.7 % of children, while for 14.3 % of children acetaminophen was self-prescribed by parents, the latter all included in group D (dose/day >90 mg/kg/day). Consequently, acetaminophen overdose was detected in 9.82 % of children, even with a medical prescription (Table 1).

As highlighted by the contingency analysis in Table 3, the caregivers’ nationality to whom acetaminophen was prescribed, was equally distributed between Italians (I) and Foreigners (F) both in group C (dose/day < 90 mg/kg/day) and in group D (dose/day > 90 mg/kg/day). No statistically significant differences have been observed, with respect to nationality, between those patients assuming doses < or > 90 mg/kg/day of acetaminophen (Chi-square test p NS). Moreover, the dose/day evaluation according to the nationality of the caregivers who administered the drug has not shown any statistically significant difference between group C and D. Also, the odds ratio analysis has not shown a significant risk with respect to caregivers’ nationality, *OR* = 0.80 (lower 95 % = 0.56, upper 95 % = 1.14)

**Table 3:** Contingency table of the nationality with respect to the daily dose of total administered acetaminophen. Nationality by group dose/day

	Group C	Group D	
Count	Daily dose	Daily dose	
Total %	<90 mg/kg/day	>90 mg/kg/day	
Col %			
Row %			
Foreing	133	51	184
	9,52	3,65	13,17
	12,55	15,13	
	72,28	27,72	
Italian	927	286	1213
	66,36	20,47	86,83
	87,45	84,87	
	76,42	23,58	
	1060	337	1397
	75,88	24,12	

Finally, a specific analysis was performed for those children who were treated with acetaminophen for body temperatures under 38 °C. These represented 21 % of the included children (293 patients), with a median age of 75 months, (a 3rd quartile of 106 months and 1 st quartile of 53 months), in whom the parent administering the drug had secondary education in 52 % of cases. Among these patients, 72 % consumed less than 90 mg/kg/day, prescribed by a doctor only in 40 % of cases. This percentage was significantly lower than patients with fever over 38 °C (Chi-square test *p* < 0.01).

## Discussion

In this study, we indicated that 74 % of caregivers start antipyretics at temperatures < 38,4 °C. Among them, 71.57 % administer it between 38 and 38.4 °C, and 21.00 % under 38 °C (Fig. 1). This attitude surely reflects a widespread fever phobia that leads to early antipyretic administration. Literature data have already described this attitude among caregivers and pediatricians [22–24], justified by the attempt of reducing the discomfort generated by fever. This therapeutic attitude is in contrast with other guidelines indicating pharmacological treatment when temperature is >40 °C or > 39 °C if the child is in discomfort [25].

Moreover about 24.12 % of our patients assumed a daily dose of acetaminophen higher than 90 mg/kg/day, of which in 97.63 % of cases a high quantity of drug was administered in a single daily dose, and not a higher frequency of administration (Table 1), as opposed to what was described in a study by James et al [26]. The assumption of higher doses of self-prescribed acetaminophen by the caregiver was 14.3 % in the studied population. This attitude of autonomous choices was more frequent in those caregivers with a higher level of education who should have better understood that acetaminophen is effectively a drug and not just an over-the-counter product, avoiding its superficial use, as evidenced by McCloskey et al [27]. This behavior has also been described in healthcare students, in which their higher knowledge on drugs seems to favor the phenomenon of self-medication [28]. Self-prescription phenomena strictly relate to the administration of high dosages of acetaminophen [1, 29].

In our study self-medication was detected in 60 % of children to whom acetaminophen was administered for body temperature <38 °C, but with doses <90 mg/kg/day in 72 % of cases.

The results of our study also highlight the preventive efficient role of the general practioners in the education for the right prescription and assumption of acetaminophen in childhood. In fact all the patients assuming a proper dose (less than 90 mg/kg/day) had a medical prescription and only 9.82 % of the studied population, assumed a dose of more than 90 mg/kg/day with a medical prescription. This evidence is in contrast with the results of other data which related the problem of acetaminophen overdose to a wrong prescription by the primary care physician [30], as to respond to the anxiety of family members, who seek an immediate resolution of their child’s fever [23].

Finally, it is important to mention the assumption of the drug as drops and syrup, (as seen in 12.03 % of the studied population), was associated with extra doses of acetaminophen, as described by other authors, who also proposed the use of simpler systems to better highlight the appropriate dosage of acetaminophen [29–32] .

## Conclusions

We believe that further interventions should be done to improve the knowledge of caregivers on the correct use of acetaminophen. Also the physiological protective role of fever, parent’s responsibility in following the pediatrician’s prescription precisely, avoiding the self-prescription phenomenon both concerning the timing to start acetaminophen and the administered doses should be emphasized in general population.
